# Supplementary material for: Neuronal ketone body utilization couples exercise and time-restricted feeding to cognitive enhancement
Source: Mol Metab. 2026 Jul 16;111:102421. doi: 10.1016/j.molmet.2026.102421 (PMC13417982; doi:10.1016/j.molmet.2026.102421)
Supplement: Supplementary file 1 [file mmc7.pdf]

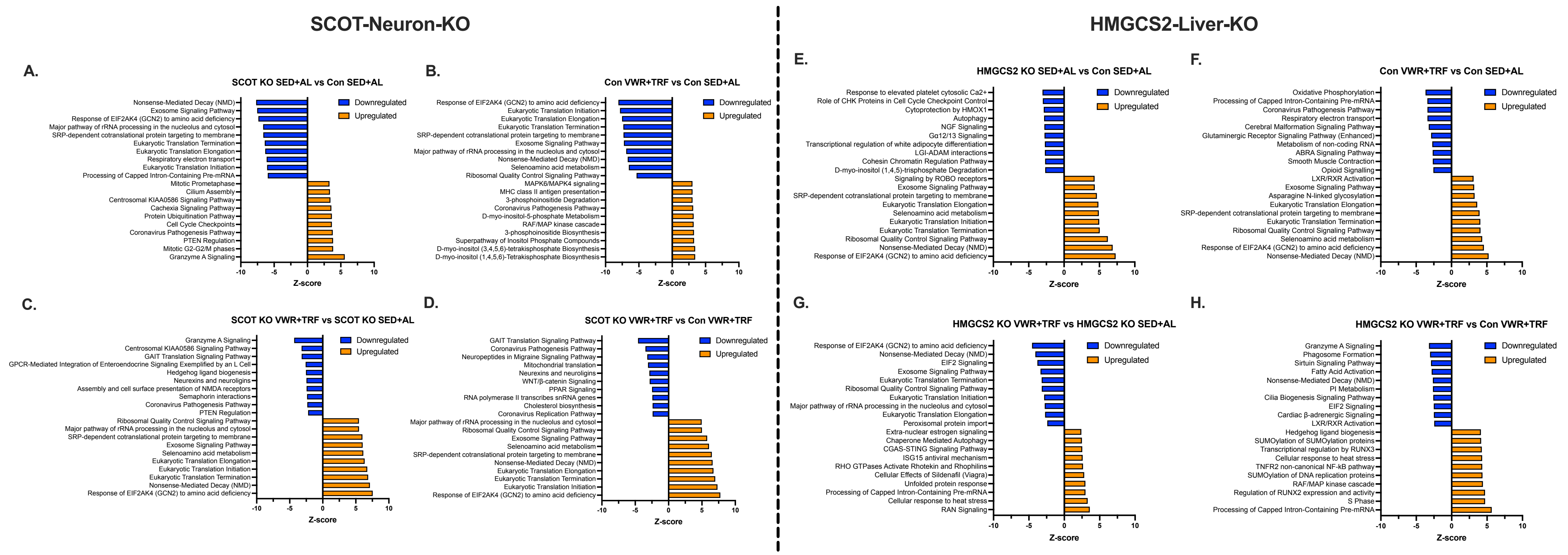
SCOT-Neuron-KO
HMGCS2-Liver-KO
A.
SCOT KO SED+AL vs Con SED+AL
Nonsense-Mediated Decay (NMD)
Exosome Signaling Pathway
Response of EIF2AK4 (GCN2) to amino acid deficiency
Major pathway of rRNA processing in the nucleolus and cytosol
SRP-dependent cotranslational protein targeting to membrane
Eukaryotic Translation Termination
Eukaryotic Translation Elongation
Respiratory electron transport
Eukaryotic Translation Initiation
Processing of Capped Intron-Containing Pre-mRNA
Mitotic Prometaphase
Cilium Assembly
Centrosomal KIAA0586 Signaling Pathway
Cachexia Signaling Pathway
Protein Ubiquitination Pathway
Cell Cycle Checkpoints
Coronavirus Pathogenesis Pathway
PTEN Regulation
Mitotic G2-G2/M phases
Granzyme A Signaling
Downregulated
Upregulated
Z-score
B.
Con VWR+TRF vs Con SED+AL
Response of EIF2AK4 (GCN2) to amino acid deficiency
Eukaryotic Translation Initiation
Eukaryotic Translation Elongation
Eukaryotic Translation Termination
SRP-dependent cotranslational protein targeting to membrane
Exosome Signaling Pathway
Major pathway of rRNA processing in the nucleolus and cytosol
Nonsense-Mediated Decay (NMD)
Selenoamino acid metabolism
Ribosomal Quality Control Signaling Pathway
MAPK6/MAPK4 signaling
MHC class II antigen presentation
3-phosphoinositide Degradation
Coronavirus Pathogenesis Pathway
D-myo-inositol-5-phosphate Metabolism
RAF/MAP kinase cascade
3-phosphoinositide Biosynthesis
Superpathway of Inositol Phosphate Compounds
D-myo-inositol (3,4,5,6)-tetrakisphosphate Biosynthesis
D-myo-inositol (1,4,5,6)-Tetrakisphosphate Biosynthesis
Downregulated
Upregulated
Z-score
C.
SCOT KO VWR+TRF vs SCOT KO SED+AL
Granzyme A Signaling
Centrosomal KIAA0586 Signaling Pathway
GAIT Translation Signaling Pathway
GPCR-Mediated Integration of Enteroendocrine Signaling Exemplified by an L Cell
Hedgehog ligand biogenesis
Neurexins and neuroligins
Assembly and cell surface presentation of NMDA receptors
Semaphorin interactions
Coronavirus Pathogenesis Pathway
PTEN Regulation
Ribosomal Quality Control Signaling Pathway
Major pathway of rRNA processing in the nucleolus and cytosol
SRP-dependent cotranslational protein targeting to membrane
Exosome Signaling Pathway
Selenoamino acid metabolism
Eukaryotic Translation Elongation
Eukaryotic Translation Initiation
Eukaryotic Translation Termination
Nonsense-Mediated Decay (NMD)
Response of EIF2AK4 (GCN2) to amino acid deficiency
Downregulated
Upregulated
Z-score
D.
SCOT KO VWR+TRF vs Con VWR+TRF
GAIT Translation Signaling Pathway
Coronavirus Pathogenesis Pathway
Neuropeptides in Migraine Signaling Pathway
Mitochondrial translation
Neurexins and neuroligins
WNT/β-catenin Signaling
PPAR Signaling
RNA polymerase II transcribes snRNA genes
Cholesterol biosynthesis
Coronavirus Replication Pathway
Major pathway of rRNA processing in the nucleolus and cytosol
Ribosomal Quality Control Signaling Pathway
Exosome Signaling Pathway
Selenoamino acid metabolism
SRP-dependent cotranslational protein targeting to membrane
Nonsense-Mediated Decay (NMD)
Eukaryotic Translation Elongation
Eukaryotic Translation Termination
Eukaryotic Translation Initiation
Response of EIF2AK4 (GCN2) to amino acid deficiency
Downregulated
Upregulated
Z-score
E.
HMGCS2 KO SED+AL vs Con SED+AL
Response to elevated platelet cytosolic Ca2+
Role of CHK Proteins in Cell Cycle Checkpoint Control
Cytoprotection by HMOX1
Autophagy
NGF Signaling
Gα12/13 Signaling
Transcriptional regulation of white adipocyte differentiation
LGI-ADAM interactions
Cohesin Chromatin Regulation Pathway
D-myo-inositol (1,4,5)-trisphosphate Degradation
Signaling by ROBO receptors
Exosome Signaling Pathway
SRP-dependent cotranslational protein targeting to membrane
Eukaryotic Translation Elongation
Selenoamino acid metabolism
Eukaryotic Translation Initiation
Eukaryotic Translation Termination
Ribosomal Quality Control Signaling Pathway
Nonsense-Mediated Decay (NMD)
Response of EIF2AK4 (GCN2) to amino acid deficiency
Downregulated
Upregulated
Z-score
F.
Con VWR+TRF vs Con SED+AL
Oxidative Phosphorylation
Processing of Capped Intron-Containing Pre-mRNA
Coronavirus Pathogenesis Pathway
Respiratory electron transport
Cerebral Malformation Signaling Pathway
Glutaminergic Receptor Signaling Pathway (Enhanced)
Metabolism of non-coding RNA
ABRA Signaling Pathway
Smooth Muscle Contraction
Opioid Signalling
LXR/RXR Activation
Exosome Signaling Pathway
Asparagine N-linked glycosylation
Eukaryotic Translation Elongation
SRP-dependent cotranslational protein targeting to membrane
Eukaryotic Translation Termination
Ribosomal Quality Control Signaling Pathway
Selenoamino acid metabolism
Response of EIF2AK4 (GCN2) to amino acid deficiency
Nonsense-Mediated Decay (NMD)
Downregulated
Upregulated
Z-score
G.
HMGCS2 KO VWR+TRF vs HMGCS2 KO SED+AL
Response of EIF2AK4 (GCN2) to amino acid deficiency
Nonsense-Mediated Decay (NMD)
EIF2 Signaling
Exosome Signaling Pathway
Eukaryotic Translation Termination
Ribosomal Quality Control Signaling Pathway
Eukaryotic Translation Initiation
Major pathway of rRNA processing in the nucleolus and cytosol
Eukaryotic Translation Elongation
Peroxisomal protein import
Extra-nuclear estrogen signaling
Chaperone Mediated Autophagy
CGAS-STING Signaling Pathway
ISG15 antiviral mechanism
RHO GTPases Activate Rhotekin and Rhophilins
Cellular Effects of Sildenafil (Viagra)
Unfolded protein response
Processing of Capped Intron-Containing Pre-mRNA
Cellular response to heat stress
RAN Signaling
Downregulated
Upregulated
Z-score
H.
HMGCS2 KO VWR+TRF vs Con VWR+TRF
Granzyme A Signaling
Phagosome Formation
Sirtuin Signaling Pathway
Fatty Acid Activation
Nonsense-Mediated Decay (NMD)
PI Metabolism
Cilia Biogenesis Signaling Pathway
EIF2 Signaling
Cardiac β-adrenergic Signaling
LXR/RXR Activation
Hedgehog ligand biogenesis
SUMOylation of SUMOylation proteins
Transcriptional regulation by RUNX3
Cellular response to heat stress
TNFR2 non-canonical NF-kB pathway
SUMOylation of DNA replication proteins
RAF/MAP kinase cascade
Regulation of RUNX2 expression and activity
S Phase
Processing of Capped Intron-Containing Pre-mRNA
Downregulated
Upregulated
Z-score
